# Elevated salivary C-reactive protein levels are associated with active and passive smoking in healthy youth: A pilot study

**DOI:** 10.1186/1476-9255-8-37

**Published:** 2011-12-07

**Authors:** Rima Azar, Annie Richard

**Affiliations:** 1 Psychobiology of Stress and Health Lab, Psychology Department, Sackville, New Brunswick, Canada; 2 University of Melbourne, Clinical Neuropsychology, Psychology Department, Melbourne, Victoria, Australia

**Keywords:** Salivary C-reactive protein, active and passive tobacco smoke exposure, cotinine

## Abstract

**Background:**

We examined *salivary *C-reactive protein (CRP) levels in the context of tobacco smoke exposure (TSE) in healthy youth. We hypothesized that there would be a dose-response relationship between TSE status and salivary CRP levels.

**Methods:**

This work is a pilot study (N = 45) for a larger investigation in which we aim to validate *salivary *CRP against serum CRP, the gold standard measurement of low-grade inflammation. Participants were healthy youth with no self-reported periodontal disease, no objectively measured obesity/adiposity, and no clinical depression, based on the Beck Depression Inventory (BDI-II). We assessed tobacco smoking and confirmed smoking status (non-smoking, passive smoking, and active smoking) with salivary cotinine measurement. We measured salivary CRP by the ELISA method. We controlled for several potential confounders.

**Results:**

We found evidence for the existence of a dose-response relationship between the TSE status and salivary CRP levels.

**Conclusions:**

Our preliminary findings indicate that salivary CRP seems to have a similar relation to TSE as its widely used serum (systemic inflammatory) biomarker counterpart.

## Background

The province of New Brunswick (NB), where this pilot study took place, has one of the highest smoking rates in Canada at 18.1% (with 19.9% of males and 16.5% of females smoking daily) [1]. Undergraduate years are a time of stress and exploration. Consequently, many youth develop permanent smoking habits, and about one-third of them will become addicted to cigarette smoking [2]. Both active and passive tobacco smoke exposure (TSE) are related to an increased risk of cardiovascular events and diseases [3,4], by altering the autonomic function or by inducing pro-inflammatory responses [4-6].

Inflammation, acute and systemic, is part of our immune reaction and leads to the release of C-reactive protein (CRP) into the bloodstream [7-9]. However, chronically increased serum CRP is a risk factor for the development of coronary artery disease (CAD) [10]. For example, chronic pro-inflammatory responses play an important role in the induction and progression of atherosclerosis [11]. Understanding the mechanisms through which TSE may affect CRP levels in young smokers is central to the prevention of early atherosclerosis. This is fundamental as CAD is one of the leading causes of death of Canadian adults. Furthermore, heart disease and stroke cost the Canadian economy more than $22.2 billion each year [10].

Serum CRP, the gold standard measurement of low-grade inflammation, can predict future cardiac events, even in healthy individuals [8]. Thus, serum CRP is often utilized to assess future CAD risk. However, serum testing is invasive in nature. In contrast, the salivary CRP assay, commercially available since November 2008 (e.g., Salimetrics Europe), is minimally invasive and yet apparently sufficiently sensitive [12,13], compared to serum CRP. It is therefore ideal for use in research on TSE in youth.

The aim of this pilot study was to investigate salivary CRP levels in the context of TSE in healthy youth. Based on findings with serum CRP [14], we tested the hypothesis of a dose-response relationship between TSE status (active, passive, non-smoking) and salivary CRP levels. Specifically, we hypothesized that salivary CRP levels would rise in a dose-response manner with the extent of TSE.

## Methods

### Study design & participants

An *a priori *power analysis indicated that a minimum of 30 participants would be sufficient to achieve power of 0.80 with an α for the ANOVA set at 0.05, and with a moderate-to-large effect size (f^2 ^= 0.50 to 0.80). To prevent or account for possible attrition/missing data, we recruited 45 healthy youth. The inclusion criterion was to be a first year university student enrolled in an "Introductory Psychology" course. Table 1 shows our exclusion criteria to determine the eligibility of interested participants. We collected demographic and health data to screen for exclusion criteria, and/or to control for potential confounders. Sixty students were approached and screened, from which 45 eligible participants (28 females and 17 males) were enrolled in the study. Their mean age was 18.89 years (*SD *= 2.62). The study was approved by the institution's Research Ethics Board. Written informed consent was obtained from the participants of this study.

**Table 1:** Exclusion Criteria for Participants

Criteria	Rationale
Periodontal disease (self-reported).	There is a positive relationship between salivary CRP and periodontal disease, which could help to explain the underlying link between periodontal disease and higher risk for CAD [27].

Obesity (based on body mass index measurement).	Obesity is a major determinant of elevated serum CRP [28].

Apparent clinical levels of depressive symptoms (screened with the Beck Depression Inventory-2^nd ^edition, BDI-II [24], which has been validated specifically with university students [25,26]).	There is increasing evidence of a relationship between depressive symptoms and elevated serum CRP, putting individuals at increased cardiac risk [8]. Depression has also been shown to be related to TSE [29].

The BDI-II is a standard measure of depression used in research that is highly correlated with the presence of symptoms of depression. The BDI-II clinical cut-off is used to indicate a high likelihood of a depressive clinical disorder.

## Materials

### TSE status

We used the Tobacco Use Questionnaire (TUQ), adapted from O'Loughlin et al. [14], which exhibited good reliability and validity with a Canadian youth population. The items were selected from a set of questions pertaining to smoking history that were used in the Québec Child and Adolescent Health Social Survey (QCAHSS); the latter were extracted from the 1994 Canadian Survey of Smoking among youths [14]. The TUQ asks about rate of smoking, type of tobacco products used, and if participants are/were exposed to tobacco smoke in their past or current living environments. We used the TUQ to identify non-smokers, passive smokers, and smokers (≥ 1 cigarette/day, on a daily basis). We confirmed TSE status with salivary cotinine measurement (Salimetrics, State College, PA, USA). The intra-assay coefficient of variation (CV) of the cotinine assay was 6.5%. The inter-assay CV was 4.21% for low values and 9.04% for high values. Cotinine high sensitivity enzyme immunoassays (EIA) were performed in duplicate. The repeated EIAs were highly correlated (r = .99, p < 0.001). Smokers had significantly higher cotinine levels (*M = *97.34, *SD *= 138.80) than passive smokers (*M *= 0.69, *SD *= 0.96), *t*(12) = 3.15, *p *= 0.009. Smokers also had higher cotinine levels than non-smokers (*M *= .83, *SD *= 1.11), *t*(12) = 3.15, *p *= 0.009. However, cotinine levels were not significantly different between passive smokers and non-smokers (p > 0.05).

### Salivary CRP levels

Salivary CRP is the main and single outcome measure of this study. Although salivary CRP does not appear to be flow rate dependent [13], flow saliva rate was determined while collecting the samples. In order to avoid any possible fluctuations in CRP levels, we tested female participants between the 4^th ^and 10^th ^day of the menstrual cycle. The saliva samples were immediately stored at -20°C until analysis (to avoid bacterial growth and loss of CRP). The concentrations of salivary CRP levels were measured by the ELISA method (Salimetrics, LLC, Pennsylvania, USA). For optimal quality-control, the same technician analyzed all samples. The % of cross-reactivity was < 0.004 for several compounds, including human albumin, IL-6, lysozyme, and alpha 1-Antitrypsin. The intra-assay CV of the CRP assay was 1.9% and the inter-assay CV was 3.7%. CRP assays were run in duplicates. Again, the repeated measures were highly correlated, *r *> .99, *p *< .001.

### Potential confounders & statistical analyses

Based on the available literature on systemic inflammation [8,15,16], we specified a priori the following potential covariates: cold/flu or infection within the past 2 weeks, systemic diseases such as diabetes mellitus, medication use such as statins, aspirin, contraceptive pills, age, body mass index (measured in kg/m^2^), non-clinical levels of depressive symptoms (BDI-II), and other substance use. The substance use was determined from the Simple Screening Instrument for Substance Abuse (SSI-SA), which is appropriate for use with youth [17]. Similarly, we established the following a priori potential covariates for cotinine measurement: intake of tea, tomatoes, potatoes and eggplants as these contain small amounts of nicotine that may contribute to the salivary cotinine levels [18]. To test our hypothesis, we compared differences in salivary CRP levels of non-smoking, passive smoking, and active smoking groups using an ANOVA and post-hoc t-tests.

## Results

**Figure 1:**
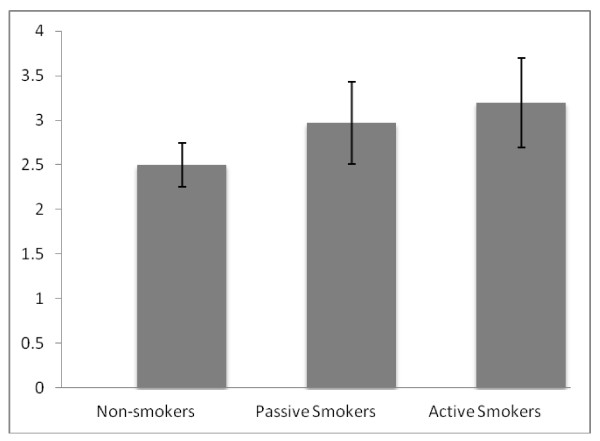
**Mean log salivary CRP (in ng/ml) for TSE status groups**. P values for comparisons with mean log salivary CRP of the non-smokers were 0.01 and 0.003 for the passive and active smoking groups respectively. Error bars indicate standard deviation.

Ten youth (22.2%) were active smokers, 22 were passive smokers (48.9%), and 13 youth (28.9%) were never smokers. The average number of daily smoked cigarettes was 4.78 (SD = 6.16). We examined salivary CRP levels in healthy youth, as a function of TSE levels. None of the potential confounders mentioned above was significantly correlated with CRP levels. Thus, we have not included them as covariates in the analyses. With an ANOVA followed by post-hoc t-tests, we compared differences in CRP levels of three groups: active smokers (smoking ≥ 1 cigarette, on a daily basis), passive smokers, and non-smokers. We found evidence for the existence of a dose-response relationship between the TSE status and salivary CRP levels (N = 45) (see Figure 1). There were significant differences between groups (*F *(2, 39) = 5.39, *p *= 0.01, *η*^2 ^= .22). Specifically, active smokers had significantly higher CRP levels (*M *= 2780.10 ng/ml, *SD *= 2501.76) than non-smokers (*M *= 371.63 ng/ml, *SD *= 255.22) (*t *(14) = 3.57, *p *= 0.00). Passive smokers (*M *= 1826.54 ng/ml, *SD *= 2744.30) also had significantly higher CRP levels than non-smokers (*t *(30) = 2.73, *p *= 0.011). Interestingly, the difference between the CRP levels of passive smokers and active smokers did not reach significance (*t *(32) = 1.24, *p *= 0.22). In addition, although salivary CRP levels were positively correlated with smoking status (r (40) = .45, p = 0.003). CRP and cotinine concentrations were not significantly correlated with each other (p > 0.05).

## Discussion

This pilot study explored the immunomodulatory effects of TSE among healthy youth, as indexed by salivary CRP. CRP levels were highest in active smokers, lower in passive smokers, and lowest in non-smokers. This supported our hypothesis of a "dose-response" effect of TSE on salivary CRP levels. This finding corroborated results of studies using the more widely used serum CRP measure [5,9,14,19]. Thus our preliminary findings suggests that salivary CRP seems to have a similar relation to TSE as its widely used serum (systemic inflammatory) biomarker counterpart [20].

A recent study [21] investigated salivary and serum CRP in healthy medical students. Although not focusing on smoking, high correlations between the two CRP biomarkers were not found. However, the study was limited by: 1. the absence of age-related reference ranges for the salivary CRP test and 2. a lack of control for periodontal disease. The latter is significant as elevated salivary CRP levels have been shown to indicate poor periodontal health or chronic oral infections (related to tissues/structures supporting the teeth). In addition, oral chronic infections have been linked to CAD [4]. More precisely, this link might be partly explained by systemic chronic inflammation [4,21], possibly in the form of subtle persistent low-grade inflammation originating from oral chronic infections.

Our study is among the very scarce literature on CRP in human saliva. To the best of our knowledge, this is the first investigation examining *salivary *CRP levels as a function of TSE levels. The strengths of this pilot study include taking into account several potential confounders. Most importantly, we ruled out the effects of reported periodontal disease on the findings (exclusion criterion). Ideally, assessing plaque induced gingivitis, while recording more objective indices of periodontal health, would have been a more precise measure of oral health. Although we have used a high-sensitivity salivary EIA assay, it is possible that this assay was not sensitive enough to capture any difference in cotinine levels between passive smokers and non-smokers. Furthermore, self-reports of undesirable social behaviours, like smoking, may be prone to under-reporting in some populations (e.g., ill older adults, pregnant women). Although under-reporting is insignificant in healthy youth, potential misclassification of smokers due to self-reporting remains a possibility. This limitation is mitigated by the use of cotinine testing. Cotinine, which is a specific, sensitive valid biomarker for determining exposure to "tobacco/nicotine", confirmed the integrity of the self-reported smoking data. Finally, different levels of physical activity, between participants in the TSE groups (active, passive), might have influenced our results. As the pilot testing did not measure physical activity, this remains a possibility that deserves future investigation [22].

## Conclusion

Inflammation may be an important physiological mechanism by which tobacco use increases risk for atherosclerosis and eventually CAD [23]. Thus, a practical use of our finding of dose-response relationship between TSE status and *salivary *CRP in young healthy smokers would be to: 1) Provide more evidence to the importance of the inflammatory pathway underlying cardiovascular risky behaviours in healthy youth and 2) Perhaps more importantly, suggest promising evidence that a simple, less invasive, & non-painful biomarker might be an alternative to the more invasive serum measure of CRP.

Our pilot finding is encouraging as it has the potential to support the future application of salivary CRP assays in populational research, and possibly eventually in clinical settings (at least in the context of young populations exposed to tobacco smoke). Further research should examine the relationship between changes in salivary CRP with serum CRP and investigate the stability of the salivary CRP biomarker over extended periods of time. These are the next steps to our pilot study. Once these steps are completed, we could assess whether the salivary CRP test has the potential to be a non-invasive and practical clinical tool to monitor risk for cardiovascular diseases in large populations.

## Competing interests

The authors declare that they have no competing interests.

## Authors' contributions

RA designed the study while AR collected and analyzed the data. Both authors contributed to writing manuscript, read and approved the final version.

## References

[B1] Statistics Canada 2009 CANSIM, table 105-0427 and Catalogue no. 82-221-X, 2009

[B2] DiFranza JR Savageau JA Fletcher K O'Loughlin J Pbert L Ockene JK McNeill AD Hazelton J Friedman K Dussault G Wood C Wellman R Symptoms of tobacco dependence after brief intermittent use: The Development and Assessment of Nicotine Dependence in youth - 2 study Arch Pediatr Adolesc Med 2007 161 704 710 10.1001/archpedi.161.7.704 17606835

[B3] Ridker PM High-sensitivity C-reactive protein, inflammation, and cardiovascular risk: From concept to clinical practice to clinical benefit Am Heart J 2004 148 19 26 10.1016/j.ahj.2004.04.028 15211329

[B4] Willershausen B Kasaj A Zahorka D Briseño B Blettner M Genth-Zotz D Münzel T Association between chronic dental infection and acute myocardial infarction J Endodontics 2009 35 626 630 10.1016/j.joen.2009.01.012 19410072

[B5] Wannamethee SG Lowe GDO Shaper AG Rumley A Lennon L Whincup PH Associations between cigarette smoking, pipe/cigar smoking, and smoking cessation, and haemostatic and inflammatory markers for cardiovascular disease Eur Heart J 2005 26 1765 1773 10.1093/eurheartj/ehi183 15817606

[B6] Hastie CE Haw S Pell JP Impact of smoking cessation and lifetime exposure on C-reactive protein Nic Tob Res 2008 10 637 642 10.1080/14622200801978722 18418786

[B7] Nordestgaard BG Zacho J Lipids, atherosclerosis and CVD risk: Is CRP an innocent bystander? Nutr Metab Cardiovasc Dis 2009 19 521 524 10.1016/j.numecd.2009.07.005 19695857

[B8] Azar R Nolan R Stewart D Listening to the heart-brain talk: persistent depressive symptoms are associated with hsCRP in apparently healthy individuals at high risk for coronary artery disease" Eur J Cardiovasc Prev Rehabilpublished online before print, July 5, 2011 10.1177/1741826711415720 21729973

[B9] Ohsawa M Okayama A Nakamura M Onado T CRP levels are elevated in smokers but unrelated to the number of cigarettes and are decreased by long-term smoking cessation in make smokers Prev Med 2005 41 651 656 10.1016/j.ypmed.2005.02.002 15917065

[B10] Canadian Heart Health Strategy and Action Plan (CHHS-AP) 2009 Ottawa 10.1016/s0828-282x(09)70116-3 PMC2732371 19668778

[B11] Clearfield MB C-Reactive Protein: A new risk assessment tool for cardiovascular disease J Am Osteopath. Assoc 2005 105 9 409 416 16239491

[B12] Ouellet-Morin I Danese A Williams B Arsenault L Validation of a high-sensitivity assay for C-reactive protein in human saliva Brain Beh Imm 2011 25 640 646 10.1016/j.bbi.2010.12.020 21236331

[B13] Salimetrics Salivary C-reactive protein ELISA kit 2009 Salimetrics cat # 1-3302, 1-3302-5

[B14] O'Loughlin J Lambert M Karper I MacGrath J Association between cigarette smoking and C-reactive protein in a representative, population-based sample of adolescents Nic Tob Res 2008 10 3 525 532 10.1080/14622200801901997 18324572

[B15] Noack B Genco RJ Grossi S Zambon JJ De Nardin E Periodontal infections contribute to elevated systemic C-reactive protein level J Periodont 2001 72 1221 1227 10.1902/jop.2000.72.9.1221 11577954

[B16] Panagiotakos DB Pitsavos C Chrysohoou C Tsetsekou E Papageorgiou C Christodoulou G Stefanadis C Inflammation, coagulation, and depressive symptomatology in cardiovascular disease-free people; the ATTICA study Eur Heart J 2004 25 492 499 10.1016/j.ehj.2004.01.018 15039129

[B17] Knight JR Goodman E Pulerwitz T DuRant RH Reliabilities of short substance abuse screening tests among adolescent medical patients Pediatr 2000 105 948 953 10742352

[B18] Siegmund B Leitner E Pfannhauser W Determination of the nicotine content of various edible nightshades (solanaceae) and their products and estimation of the associated dietary nicotine intake J Agric Food Chem 1999 47 3113 3120 10.1021/jf990089w 10552617

[B19] Fröhlich M Sund M Löwel H Imhof A Hoffmeister A Koenig W Independent association of various smoking characteristics with markers of systemic inflammation in men: Results from a representative sample of the general population (MONICA Augsburg survey 1994/95 Eur Heart J 2003 24 1365 1372 10.1016/S0195-668X(03)00260-4 12871694

[B20] Janeway CA Travers P Jr Walport M Shlomchik MJ Immunobiology: The Immune System in Health and Disease 2005 6 New York: Garland Publishing

[B21] Dillon MC Opris DC Lickliter J Cornwell HN Bridges EG Nazar AM Bridges KG Detection of Homocysteine and C-Reactive Protein in the Saliva of Healthy Adults: Comparison with Blood Levels Biomark Insights 2010 5 57 61 20703322 10.4137/bmi.s5305 PMC2918353

[B22] Tremblay MS Shephard RJ McKenzie TL Gledhill N Physical activity assessment options within the context of the Canadian Physical Activity, Fitness, and Lifestyle Appraisal Can J App Physiol 2001 26 4 388 407 10.1139/h01-024 11487710

[B23] He J Vupputuri S Whelton PK Relationship between cigarette smoking and novel risk factors for cardiovascular disease in the United States Ann Intern Med 2003 138 891 897 12779299 10.7326/0003-4819-138-11-200306030-00010

[B24] Beck AT Steer RA Garbin MG Psychometric properties of the Beck Depression Inventory: twenty-five years of evaluation Clin Psychol Rev 1988 8 77 100 10.1016/0272-7358(88)90050-5

[B25] Bouteyre E Maurel M Bernaud JL Daily hassles and depressive symptoms among first year psychology students in France: the role of coping and social support Stress Health 2007 23 2 93 99 10.1002/smi.1125

[B26] Lipps GE Lowe GA Young R Validation of the beck depression inventory-II in a Jamaican university student cohort West Ind Med J 2007 56 5 404 8 18303751

[B27] Slade GD Offenbacher S Beck JD Heiss G Pankow JS Acute-phase inflammatory response to periodontal disease in the US Population J Dent Res 2000 79 49 57 10.1177/00220345000790010701 10690660

[B28] Wee CC Mukamal KJ Huang A Davis RB McCarthy EP Mittleman MA Obesity and C-reactive protein levels among White, Black, and Hispanic US adults Obesity 2008 16 875 880 10.1038/oby.2008.7 18379563 PMC2848449

[B29] Cardiovascular Risk in Young Finns Study Elovainio M Keltikangas-Järvinen L Pulkki-Råback L Kivimäki M Puttonen S Viikari L Räsänen L Mansikkaniemi K Viikari J Raitakari OT Depressive symptoms and C-reactive protein: The cardiovascular risk in Young Finns Study Psychol Med 2006 36 797 805 10.1017/S0033291706007574 16623962

